# Development of Postoperative Pain in Patients with End-Stage Knee Osteoarthritis Is Associated with Upregulation of Genes Related to Extracellular Matrix Degradation, Inflammation, and Apoptosis Measured in the Peripheral Blood before Knee Surgery

**DOI:** 10.3390/life10100224

**Published:** 2020-09-30

**Authors:** Elena V. Tchetina, Kseniya E. Glemba, Galina A. Markova, Evgeniy A. Naryshkin, Elena A. Taskina, Maksim A. Makarov, Aleksandr M. Lila

**Affiliations:** 1 Immunology and Molecular Biology Department, Nasonova Research Institute of Rheumatology, 115522 Moscow, Russia; g.markova2010@yandex.ru; 2 Surgery Department, Nasonova Research Institute of Rheumatology, 115522 Moscow, Russia; kseniyaglemba@yandex.ru (K.E.G.); naryshkin.evgeniy@gmail.com (E.A.N.); ortopedniir@mail.ru (M.A.M.); 3 Osteoartritis Laboratory, Nasonova Research Institute of Rheumatology, 115522 Moscow, Russia; braell@mail.ru (E.A.T.); amlila@mail.ru (A.M.L.)

**Keywords:** knee osteoarthritis, postoperative pain prediction, gene expression, peripheral blood

## Abstract

Osteoarthritis (OA) pain implies an indication for joint replacement in patients with end-stage OA. However, chronic postoperative pain is observed in 10–40% of patients with OA. Here, we identified genes whose expression in the peripheral blood before surgery could denote the risk of postoperative pain development. We examined the peripheral blood of 26 healthy subjects and 50 patients with end-stage OA prior to joint replacement surgery. Pain was evaluated before surgery using the visual analog scale (VAS) index and neuropathic pain questionnaires, Douleur Neuropathique 4 Questions (DN4) and PainDETECT questionnaires. Functional activity was assessed using the Western Ontario and McMaster Universities osteoarthritis index (WOMAC). Three and six months after surgery, pain indices according to VAS of 30% and higher were considered. Metalloproteinase (MMP)-9 and tissue inhibitor of metalloproteinase (TIMP)1 protein levels were measured using ELISA in the peripheral blood mononuclear cells (PBMCs). Total RNA isolated from whole blood was analysed using quantitative real-time RT-PCR for caspase-3, MMP-9, TIMP1, cathepsins K and S, tumour necrosis factor (TNF)α, interleukin (IL)-1β, and cyclooxygenase (COX)-2 gene expression. Seventeen patients reported post-surgical pain. Expression of cathepsins K and S, caspase-3, TIMP1, IL-1β, and TNFα genes before surgery was significantly higher in these patients compared to pain-free patients with OA. Receiver-operating characteristic (ROC) curve analyses confirmed significant associations between these gene expressions and the likelihood of pain development after arthroplasty. High baseline expression of genes associated with extracellular matrix destruction (cathepsins S and K, TIMP1), inflammation (IL-1β, TNFα), and apoptosis (caspase-3) measured in the peripheral blood of patients with end-stage OA before knee arthroplasty might serve as an important biomarker of postoperative pain development.

## 1. Introduction

Osteoarthritis (OA) is a systemic disease that involves single or multiple joints. It produces articular cartilage degradation, remodelling of the subchondral bone, and is associated with synovial inflammation [1]. Pain in OA is the main clinical symptom that limits a patient’s working capacity and everyday self-care. Because there are currently no disease-modifying drugs for OA therapy, the treatment comprises of pain control using basic anti-inflammatory drugs (NSAIDs), glucocorticoids, and chondroprotectors. Severe pain is one of the most important indications for joint replacement in patients with end-stage OA. Knee joint arthroplasty is the most common OA treatment worldwide, with an annual increase in the number of cases, and it is inspected expected to increase seven-fold by 2030 [2]. Meanwhile, pain after knee replacement surgery persists in 10–40% of patients [3]. Therefore, identification of the causes that affect the outcome of arthroplasty would permit a more accurate selection of patients, provide them with more accurate anticipations, and augment the profits of surgery for each individual [4]. In addition, there is a need to predict the results of surgical intervention in terms of maintaining postoperative pain due to the high cost of arthroplasty. 

In OA, pain is classified as (1) nociceptive, which occurs when damage or inflammation, or both, of the joint tissues is induced by activation of tissue nociceptors and ensures a protective function, or (2) neuropathic, which is caused by the damage or dysfunction of the nervous system and includes impaired peripheral and central sensitization mechanisms [5,6]. Peripheral sensitization is designated by a decrease in threshold values and amplification of nociceptor sensitivity [7]. Central sensitization is characterized by an excessive response of central neurons to receptor signals. It includes an altered signal transmission from sensory neurons, impaired function of descending anti-nociceptors, increased activity of pain-enhancing paths, temporal summation (wind-up), and long-term potentiation of neural synapses in the cerebral cortex [8]. Recent studies have suggested that the putative causes for the pain persistence after arthroplasty could result from a predominance of central sensitization in 30% of patients with OA, including subjects with end-stage OA and in those who used opioids or antidepressants before surgery [9]. Therefore, it was proposed to use methods of temporal summation (wind-up) or conditional pain modulation (CPM) as predictors of postoperative pain [10,11,12]. Further studies proposed the assessment of pain using quantitative sensory testing (QST) because the dysfunction in the descending pain modulatory system was associated with the dysfunction in peripheral sensory neurons [13]. However, systematic analyses have revealed that QST prior to surgery does not reliably predict the postoperative pain [14]. 

Postoperative chronic pain is also associated with a patient’s sociodemographic and clinical factors (female gender, older age, higher levels of baseline pain), as well as with predictive psychological factors (depression and anxiety) [15], metabolic disorders, such as high body mass index (BMI), obesity, inflammation, comorbidity [16], and arthritis of other joints [17]. However, putative prognostic factors, based on the associations between significant determinants measured before arthroplasty and the results of surgery, failed to predict the postoperative pain unequivocally [18].

Recent studies of molecular mechanisms of pain have revealed a number of molecular markers, which include some cytokines, chemokines, calcium or glutamate transporters, caspases, and proteases [19]. In a single study, it was demonstrated that high protein concentrations of tumour necrosis factor (TNF) α, metalloproteinase (MMP)-13, and IL-6 in the synovial fluid were independent predictors of postoperative pain 2 years after arthroplasty [20]. These results hold a promise of using some of these gene expressions as predictors of potential postoperative complications before surgery. However, synovial fluid is not easily accessible outside surgery.

Therefore, this preliminary prospective study aimed to identify genes whose expressions in the peripheral blood before knee arthroplasty could be associated with the post-surgical pain development in patients with end-stage OA.

## 2. Materials and Methods

### 2.1. Patients

The inclusion criteria for control subjects: 26 individuals (average age 65.8 ± 7.3 years, range 42–74 years) who were free from any serious illnesses and were recruited from the Moscow area. The control subjects were of comparable age to the examined patients with end-stage OA. The exclusion criteria for control subjects included any degree of knee pain. 

The inclusion criteria for patients with end-stage OA: 50 unrelated subjects with primary OA of the knee who underwent primary total knee replacement surgery at the Nasonova Research Institute of Rheumatology between March 2018 and June 2019. The average age of patients with OA was 67.6 ± 7.5 years (range 54–82 years). These patients had radiographic Kellgren and Lawrence (K&L) OA grades of III–IV, experienced constant pain during the last three months, and had walking problems (lameness). The exclusion criteria: decompensated chronic diseases, active infectious process and foci of chronic infection, neurocirculatory disorders of the lower extremities, opioid-type analgesic therapy prior to surgery.

The following NSAIDS were used as pain medications: meloxicam (15 mg/day) (n = 2), nimesulide (200 mg/day) (n = 22) or diclofenac (200 mg/day) (n = 11), etoricoxib (60 mg/day) (n = 4), ketoprofen (100–200 mg/day) (n = 2), and celecoxib (200 mg/day) (n = 2). Patients were also treated with the symptomatic slow-acting drugs for osteoarthritis (SYSDOA) such as chondroitin sulphate (800 mg/day) (n = 10), and glucosamine sulphate (1500 mg/day) (n = 12). Medium molecular weight hyaluronate (2 mL, 3–5 doses/week) (n = 15) and a steroid bethametasone (1 mL) (n = 13) were also applied.

All of the patients with end-stage OA fulfilled the criteria of the American College of Rheumatology regarding OA [21]. The exclusion criteria for OA patients were any type of previous knee surgery; rheumatoid arthritis; systemic inflammatory joint diseases, secondary arthritis associated with reactive arthritis, gout, pseudogout, intraarticular fractures, ochronosis, acromegaly, Wilson disease, Padgett’s disease, primary synovial chondromatosis, hemochromatosis, chondrocalcinosis, aseptic necrosis of femoral or tibia condilas, and other abnormalities including renal diseases; thyroid, parathyroid or other endocrinological diseases; diabetes mellitus; uncontrolled arterial hypertension; instable angina; gastric or duodenal ulcer; vascular insufficiency; bleeding; or thrombophlebitis. Women who had taken drugs such as estrogen, progesterone, bisphosphonates, glucocorticoids, and alfacalcidol were not included in the study.

All total knee replacements were performed under spinal anaesthesia by a surgical team comprising two orthopaedic surgeons. The standard protocol was used for surgery, including a midline skin cut, a standard medial parapatellar method and a measured resection technique. Intravenous tranexamic acid was applied 5 to 10 min before the skin incision (20 mg/kg) and 3, 6, 12, and 24 h later (10 mg/kg) along with 1 g of topical tranexamic acid in 50 mL of normal saline solution. All patients were evaluated by a physician 3 times daily until hospital dismissal. Walking with partial weight-bearing and a knee brace to protect the surgical locus was advised on the second day after surgery. To prevent deep venous thromboembolism (DVT), all patients used graduated compression stockings. In addition, a full-dose of nadroparin was prescribed daily until discharge. If no bleeding events occurred, 10 mg of rivaroxaban was recommended orally for 14 days after discharge. All patients were discharged 7 days after surgery. Doppler ultrasound was used to evaluate for DVT periodically during 3-month follow-up.

### 2.2. Clinical Testing 

Patient demographics, including age and sex, were recorded. The radiographic grade of OA was determined by the analysis of the weight-bearing anteroposterior radiographs of the knees and was counted according to Kellgren and Lawrence [22]. The Western Ontario and McMaster Universities osteoarthritis index (WOMAC) visual analogue scale was used to evaluate pain, stiffness, and physical function [23]. Nociceptive pain was evaluated using the visual analogue scale (VAS), whereas neuropathic pain was measured using PainDETECT [24] and DN4 (Douleur Neuropathique en 4 Questions) [25] questionnaires. The Brief Pain Inventory (BPI) questionnaire was used for assessment of pain severity [26]. The Hospital Anxiety and Depression Scale (HADS) was used to reveal the levels of anxiety and depression among patients with OA [27].

### 2.3. Quantification of MMP-9 and TIMP1 Protein Levels

Peripheral blood (10 mL) was gathered in vacutainers containing ethylenediaminetetraacetic acid (EDTA) (BDH, England). The blood samples were taken in a standard manner between 07:00 a.m. and 09:00 a.m. Whole blood was fractionated in a Ficoll density gradient. Peripheral blood mononuclear cells (PBMCs) from the interphase were collected and washed twice in phosphate-buffered saline [28]. The obtained PBMCs were frozen and kept at −80 °C until protein extraction.

Concentrations of MMP-9 (BMS2016-2) and TIMP1 (BMS2018) were determined in isolated PBMCs using commercially available enzyme-linked immunosorbent assay (ELISA) kits (Bender MedSystems GmbH, Vienna, Austria) according to the manufacturer’s instructions. Results were expressed per µg of DNA measured in PBMC lysates. PBMC lysates were acquired using Cell Extraction Buffer containing 10 mM Tris, pH 7.4, 100 mM NaCl, 1 mM EDTA, 1 mM EGTA, 1 mM NaF, 20 mM Na_4_P_2_O_7_, 20 mM Na_3_VO_4_, 1% Triton X-100, 10% glycerol, 0.1% SDS, and 0.5% deoxycholate (Invitrogen, Camarillo, CA, USA) supplemented with Protease Inhibitor Cocktail (Sigma-Aldrich, Inc, St. Louis, MO, USA) and 1 mM PMSF (Sigma-Aldrich, Inc, St. Louis, MO, USA) according to the manufacturer’s instructions. Total DNA content in PBMC lysates was measured spectrophotometrically using a GeneQuant device (Amersham Biosciences, Watertown, USA). Results were expressed per µg of DNA. 

### 2.4. Total RNA Isolation and Reverse Transcriptase (RT) Reaction

Total RNA was isolated from 100µl of whole blood immediately after withdrawal using Extract RNA reagent (Evrogen, Moscow, Russia) in accordance with the manufacturer’s recommendations. Total RNA had an A_260/290_ > 1.9. The RT-reaction was performed using a M-MLV RT kit containing Moloney Murine Leukemia Virus (M-MLV) Reverse Transcriptase, random hexanucleotide primers and total RNA according to the manufacturer’s recommendations (Evrogen, Moscow, Russia).

### 2.5. Real-Time Quantitative PCR

The TaqMan primers and probes (Applied Biosystems, Foster City, CA, USA) for expression of human genes cathepsin S (Hs00175407_m1), cathepsin K (Hs00166165_m1), caspase 3 (Hs00263337_m1), TNFα (Hs00174128_m1), IL-1β (Hs00174097_m1), COX-2 (Hs00153133_m1), MMP-9 (Hs00234579_m1) and TIMP1(Hs00171558_m1) were used. *β-Actin* served as an endogenous control. 

Quantification of gene expression was conducted using a Quant Studio 5 Real-Time PCR System (Applied Biosystems, Foster City, CA, USA). A volume of 1 μL of RT product was subjected to real-time PCR in a 15 μL total reaction mixture including 7.5 μL of TaqMan Universal PCR Master Mix (Applied Biosystems), 900 nM sense and antisense primers, 50 nM probe, and cDNA template. After a first step of 50 °C for 2 min and initial warming at 95 °C for 10 min, reaction mixtures were exposed to 40 amplification cycles (15 s at 95 °C for denaturation and 1 min of annealing and extension at 60 °C) [29].

Relative mRNA expression was determined using the delta-delta C_T_ method, as described by the manufacturer (Applied Biosystems) [30]. The delta C_T_ value was calculated by subtracting the C_T_ value for the housekeeping gene β-Actin from the C_T_ value for each sample. A delta-delta C_T_ value was then calculated by subtracting the deltaC_T_ value of the control (each healthy patient) from the delta C_T_ value of each OA patient. Each PCR was performed in duplicate. Three “no template” controls were consistently negative for each reaction. 

**Figure 1:**
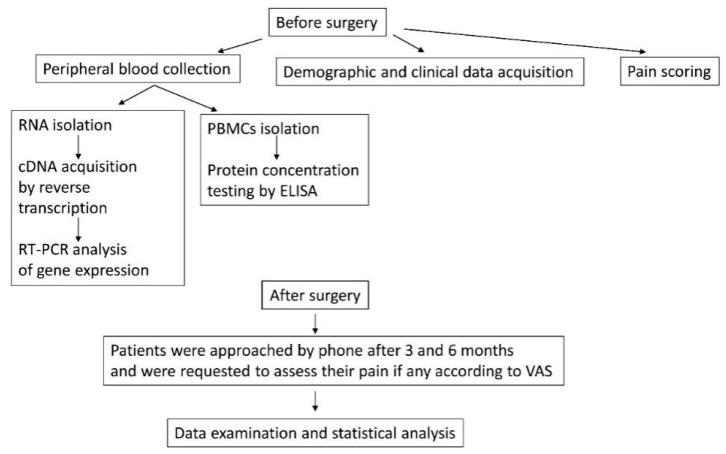
Diagram of the steps and measurements made in the study.

All steps and measurements made in the study are presented at Figure 1. 

### 2.6. Statistical Analysis 

A Kolmogorov-Smirnov and Shapiro-Wilk normality tests were applied for data distribution analysis. Spearman’s rank correlations and Mann-Whitney U-test were used to non-normally distributed data which was expressed as median (quartiles). The receiver operating characteristic (ROC) curve analyses are presented as the areas under the curve (AUCs) and the 95% confidence intervals (CIs). The diagnostic efficacies of the gene expression values were assessed using the sensitivities and specificities at the cut-off points. Logistic regression was used to model the relationship between expression of the examined genes prior to surgery and postoperative pain preservation six months after surgery. The logistic regression model was built using the stepwise inclusion method. To compare percentages, a two-tailed Z-test for percentages was applied. Statistica for Windows and Statistical Package for the Social Sciences (SPSS) version 19 software (IBM, Armonk, NY, USA) were used for all of the statistical analyses. *p*-values ≤ 0.05 were considered significant. 

### 2.7. Ethical Approval

The study protocol was approved by the Local Committee on the Ethics of Human Research and informed consent was obtained from all subjects (Protocol No. 32 from 20 December 2018).

## 3. Results

### 3.1. Clinical Parameters of the Examined Patients with End-Stage OA before Surgery

Analysis of the demographic and clinical characteristics of 50 patients of both genders with end-stage OA revealed that the K&L OA grades of the examined subjects varied from III to IV (grade III, 37 patients; grade IV, 13 patients). The average age of these patients was 67.6 years (range 54–82 years), whereas the average disease duration was 9.9 years (range 3–30 years). The majority of patients demonstrated an increased Body Mass Index (BMI) average 30.5 (range 21.3–39.6). The total WOMAC scoring was estimated as 1065 (range 300–1435), whereas average total pain was 222.8 (range 70–360), total physical function, 746 (range 360–1040), and total stiffness, 93.9 (range 40–130).

All patients complained of permanent pain during the last three months. Pain scoring according to VAS revealed moderate rates of knee pain of 64.4 (range 20–90) in these patients with OA. According to the Brief Pain Inventory (BPI) questionnaire, the average pain severity was 4.8 (range 2.3–6.3). Neuropathic pain evaluation using the DN4 questionnaire had an average score of 1.9 (range 1–7) and revealed only one patient with a score of seven. The average score according to the PainDETECT questionnaire was 6.0 (range 1–18), indicating no patient with neuropathic pain. According to Hospital Anxiety and Depression Scale (HADS), abnormal anxiety was observed in three patients (with the scores of 11 and 16) and 10 patients out of 50 exhibited borderline levels (scores 8–10). In addition, 8 of 50 patients were depressed because they had a total score >11, whereas 14 of 50 patients had borderline values (scores 8–10) according to the HADS depression scale.

### 3.2. Baseline Clinical Parameters of the Examined OA Subjects, Who Developed Post-Surgical Pain after 6 Months Compared to Pain-Free Patients

Patients were interviewed about pain sensation according to VAS after 3 and 6 months post-surgery. Post-surgical pain complaints at both time points were observed in 17 (34%) out of 50 patients. The average pain levels reported after surgery were 34.3 ± 2.3% (3 months) and 38.6 ± 2.5% (6 months) (range from 30% to 50%) according to VAS, and they did not differ significantly (*p* = 0.26). Average pain improvement in patients who developed pain after surgery did not exceed 20%. 

Comparison of clinical traits in both examined subsets of patients with OA revealed essentially similar baseline levels for the majority of indices (Table 1). However, a trend of higher BPI pain severity (*p* = 0.07) prior to surgery was observed in patients who were satisfied with the surgery. In addition, patients who developed post-surgical pain had more often arterial hypertension.

### 3.3. Whole Blood Gene Expression 

**Figure 2:**
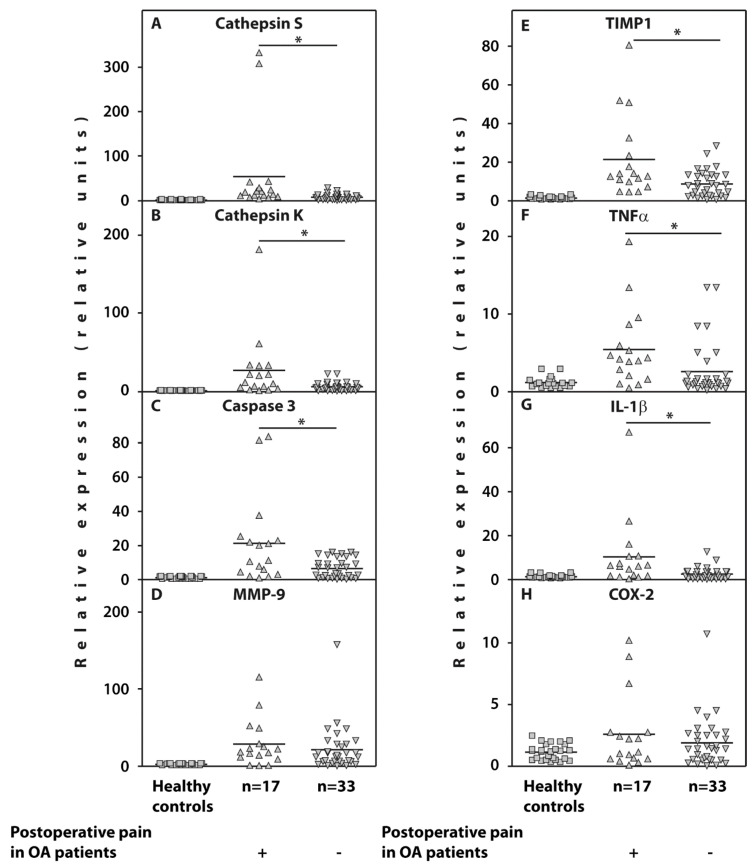
Relative expression of the genes cathepsin S (**A**), cathepsin K (**B**), caspase-3 (**C**), MMP-9 (**D**), TIMP1 (**E**), TNFα (**F**), IL-1β (**G**), and COX-2 (**H**) related to *β-actin* determined by real-time PCR analyses in the whole blood of end-stage OA patients who either developed postoperative pain (n = 17) (positive triangles) or were satisfied by surgery results (n = 33) (inverted triangles) compared to healthy controls (n = 26) (squares). Controls are shown as 1.0 as required for relative quantification with the real-time PCR protocol. Asterisks (*) indicate significant differences (Mann-Whitney U-test) between examined subsets of patients with end-stage OA.

Examination of gene expression in the whole blood of the examined patients with end-stage OA revealed a significant upregulation of cathepsin S and cathepsin K, caspase-3, TIMP1, IL-1β, and TNFα in those who developed post-surgical pain compared to 33 pain-free subjects (Table 2; Figure 2). 

However, the gene expression of MMP-9 and COX-2 was not significantly different in both cohorts of patients with OA.

### 3.4. Protein Levels of MMP-9 and TIMP1 in Isolated PBMCs 

**Figure 3:**
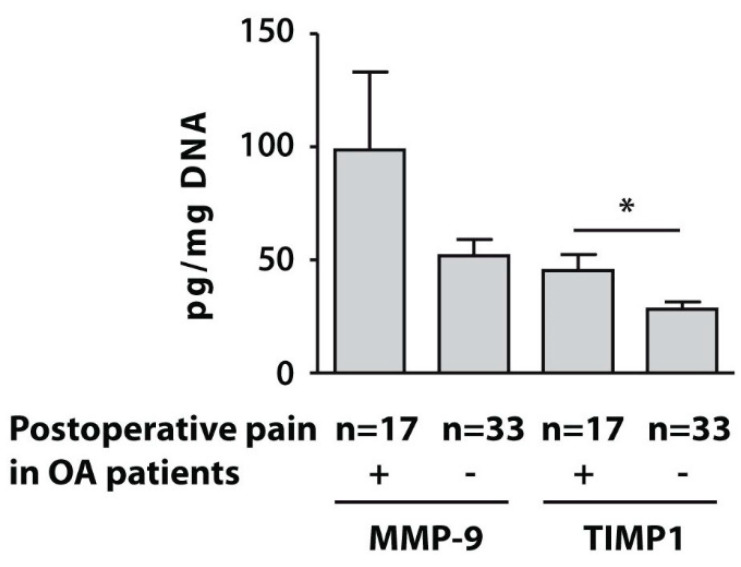
Protein concentrations of MMP-9 and TIMP1 measured by ELISA in PBMCs from patients with end-stage OA who developed postoperative pain (n = 17) and pain-free subjects (n = 33). Asterisk (*) indicates significant differences (Mann-Whitney U-test) between examined subsets.

To assess the clinical significance of relative expression of the examined genes in the whole blood of the subjects with end-stage OA, we analysed the protein concentrations of MMP-9 and TIMP1 in the PBMC fraction. The protein levels of TIMP1 in the examined seventeen end-stage patients with OA who developed post-surgical pain was also significantly higher, compared to pain-free subjects, while MMP-9 protein concentrations were not significantly different between the examined subsets (Figure 3).

### 3.5. Correlation Analyses of the Gene Expressions with Clinical and Radiographic Parameters

Bivariate correlation analyses using Pearson Spearman’s correlation coefficients for normally distributed data and Spearman’s correlation coefficients for the remaining results have revealed a positive correlation between the K&L radiological grade, PainDETECT index, and pain at night, although its baseline scores were moderate, 33.0 (range 10–80) (Table 3). BPI pain severity and HADS anxiety scores positively correlated with total pain, total physical function and total WOMAC indices. In addition, neuropathic pain scores based on DN4 and PainDETECT questionnaires, have revealed a positive correlation with each other and HADS depression. Bivariate correlation analyses using Spearman’s correlation coefficients of the expressions of the examined genes with the clinical and radiographic parameters at baseline in all the examined patients with OA (n = 50) revealed a positive correlation of BMI values with cathepsin S (r = 0.307, *p* = 0.03), cathepsin K (r = 0.335, *p* = 0.01), caspase 3 (r = 0.307, *p* = 0.03), MMP-9 (r = 0.439, *p* = < 0.01), and TIMP1 (r = 0.329, *p* = 0.02) gene expression. TIMP1 gene expression also negatively correlated with total pain (r = −0.317, *p* = 0.02) and total WOMAC (r = −0.290, *p* = 0.04) indices. Neuropathic pain-related DN4 scores positively correlated with TNFα (r = 0.330, *p* = 0.02) and IL-1β (r = 0.496, *p* = <0.01) gene expression. IL-1β gene expression also positively correlated with PainDETECT scores (r = 0.313, *p* = 0.04).

**Figure 4:**
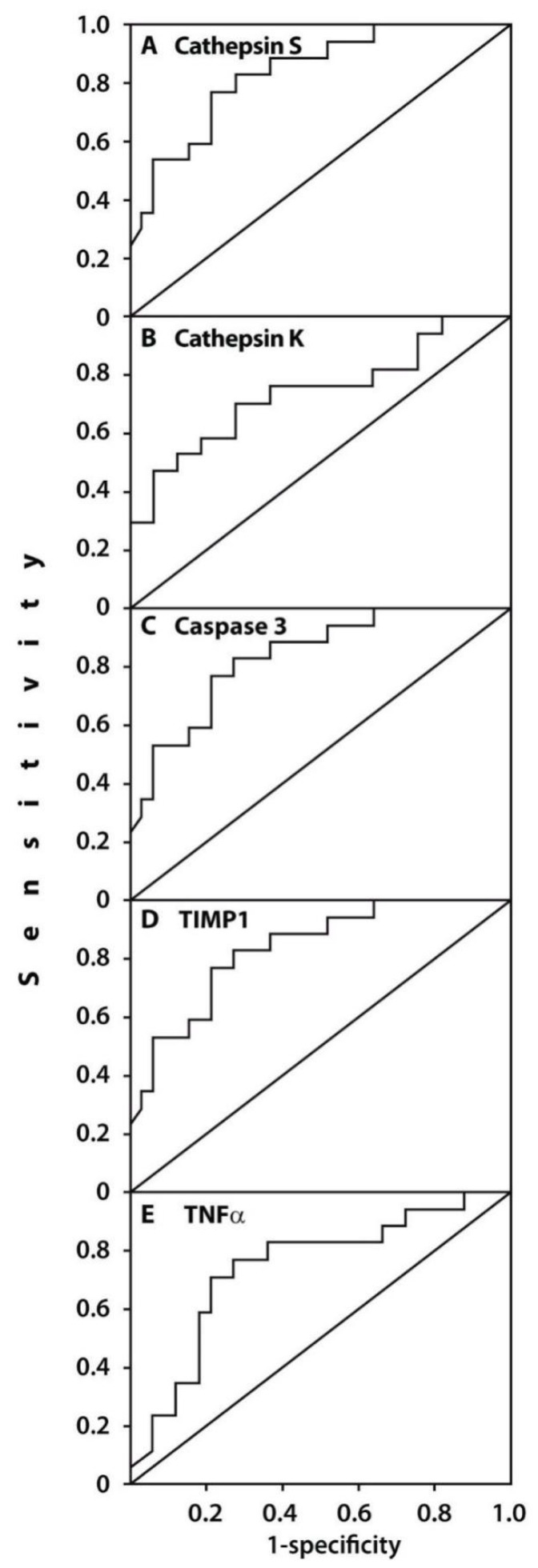
Areas under the curve (AUCs) between the baseline gene expressions in the peripheral blood of patients with end-stage OA who developed postoperative pain (n = 17) and pain-free subjects (n = 33) (**A**–**F**). Receiver-operating characteristic (ROC) curves for the expressions of (**A**) cathepsin S (AUC = 0.835, 95% CI (0.721–0.949), *p* = 0.000), (**B**) cathepsin K (AUC = 0.743, 95% CI (0.589–0.898)), *p* = 0.005), (**C**) caspase 3 (AUC = 0.732, 95% CI (0.577–0.886), *p* = 0.008), (**D**) TIMP1 (AUC = 0.741, 95% CI (0.603–0.879), *p* = 0.006), and (**E**) TNFα (AUC = 0.738, 95% CI (0.589–0.887), *p* = 0.006).

The prognostic values of these gene expressions were assessed using ROC curve analyses (Figure 4), which confirmed a potential diagnostic/predictive value of expressions of the examined genes before surgery with the likelihood of pain development after surgery. The cut-off values for the examined gene expressions were 9.09 for cathepsin S (AUC = 0.835, 95% CI (0.721–0.949), *p* = 0.000, sensitivity of 0.82% and specificity of 0.73%), 7.67 for caspase 3 (AUC = 0.732, 95% CI (0.577–0.886), *p* = 0.008, sensitivity of 0.65% and specificity of 0.64%), 5.96 for cathepsin K (AUC = 0.743, 95% CI (0.589–0.898), *p* = 0.005, sensitivity of 0.71% and specificity of 0.67%), 1.88 for TNFα (AUC = 0.738, 95% CI (0.589–0.887), *p* = 0.006, sensitivity of 0.76% and specificity of 0.73%), 3.3 for IL-1β (AUC = 0.763, 95% CI (0.615–0.910), *p* = 0.003, sensitivity of 0.65% and specificity of 0.65%), and 9.12 for TIMP1 (AUC = 0.741, 95% CI (0.603–0.879), *p* = 0.006, sensitivity of 0.67% and specificity of 0.64%). 

Logistic regression modeling demonstrated that high expression of caspase 3 (*p* = 0.014), cathepsin K (*p* = 0.011), cathepsin S (*p* = 0.015), IL-1β (*p* = 0.011), TIMP1 (*p* = 0.05), and TNFα (*p* = 0.01) were independent predictors of postoperative pain development. However, these results require further studies due to the small number of patients in both subsets. 

## 4. Discussion

Several strong independent predictors for pain persistence after knee replacement surgery have been identified, including pain catastrophizing, mental health, preoperative knee pain, and pain at other sites [31]. However, other studies found that pre-surgical values of clinical and biopsychosocial variables were not predictive for postoperative pain because pain is a subjective sensation and includes physiological, cognitive, and emotional components [32]. 

Here we suggest using the baseline peripheral blood expression of genes associated with extracellular matrix turnover (cathepsins S and K, and TIMP1), apoptosis (caspase-3), and inflammation (TNFα and IL-1β) as prognostic markers of post-surgical pain. In our study group, these genes were expressed significantly higher before surgery in those patients with end-stage OA who developed post-surgical pain compared to OA subjects who were satisfied with the surgery results. The high predictive values of the abovementioned gene expressions in the development of pain after knee replacement surgery were confirmed by the ROC curve profiles for expressions of these genes and could be used in clinical settings for prediction of post-surgical pain development. 

Our results are in line with previous observations stating that increased concentrations of cytokines in the synovial fluid both correlated with pain in the early stages of OA [33] and were independent predictors of lesser pain improvement after knee surgery [20]. However, we noted that not every cytokine gene expression could serve as a pain prognostic marker, as in the examined OA patient cohort COX-2 baseline gene expression did not significantly differ between both subsets. Previous studies showed that increased activity of MMP-9 involving an inflammatory response due to proteolytic maturation of cytokines contributed to increased pain-related behaviour in response to injury that can be reversed by TIMP1 activity [34]. Therefore, significant upregulation of TIMP1 gene expression observed in patients with end-stage OA who developed pain after surgery in our study, as well as negative correlation between TIMP1 gene expression and pain indices, might further point to a high and uncontrollable overall gene expression of MMPs and proinflammatory cytokines, which are responsible for pain maintenance. Furthermore, since the pain-sensing mechanism of cathepsin S involves the interruption of T-cell activation and peripheral cytokine release [35], its upregulation before surgery in the examined patients with end-stage OA who developed post-surgical pain is reasonable. Upregulation of cathepsin K gene expression associated with postoperative pain might be due to the increased mechanosensitivity of knee afferent nerve activity, which was recently demonstrated in animal OA studies [36], whereas caspase-3 upregulation in the same patients might result from the enhanced pain behaviour due to activation of sensory neuronal subsets, which was previously observed in diabetic patients [37].

Considering baseline clinical and psychosocial variables of the examined subjects with end-stage OA, our observations support the results of previous studies. For example, the examined patients with OA demonstrated essentially similar traits in both examined subsets according to radiographic K&L grade, the disease duration, ESR values, and WOMAC scores. This finding indicates that in our cohort the level of sensitivity for clinical determinants as predictive factors for the postoperative pain development is not sufficient. Similar observations were also noted by others [38], although some studies demonstrated an association between a lower preoperative status and a worse outcome [18]. Moreover, a positive correlation of baseline DN4 indices with TNFα and IL-1β gene expression in the peripheral blood might indicate that neuropathic pain symptoms are exacerbated in the inflammatory milieu, as was suggested previously [39]. In addition, the greater incidence of comorbidities, such as arterial hypertension, was associated with a worse outcome of knee surgery in the examined patients with OA, as was also observed before [40]. Our results are in line with previous studies, which have also shown that obesity was among the strongest predictors of increased postoperative use of analgesics [41] and can produce negative effects on prosthesis survival [15]. 

Furthermore, the psychological factors, primarily depression, were previously found to have the greatest effect on postoperative pain [18] and are supported by our results. Therefore, it is important to assess the incidence of this condition before surgery. The positive correlation of pain indices and DN4 scores with PainDETECT, HADS depression/anxiety, and BPI pain severity variables, as observed in our study, indicates the association of neuropathic pain symptoms with pain intensity in the preoperative time, and it has also been suggested previously [13]. Our observation of slightly higher BPI pain severity prior to surgery in patients who were satisfied with the surgery is supported by previous observations indicating that patients with severe OA have significantly more improvement in their usual activities and pain after knee surgery than patients with less severe OA [42].

## 5. Conclusions

In summary, our study demonstrated that high baseline expression of genes associated with extracellular matrix degradation (cathepsins S and K, and TIMP1), inflammation (TNFα and IL-1β), and apoptosis (caspase-3) measured in the peripheral blood of patients with end-stage OA before knee replacement surgery might serve as important biomarkers of postoperative pain development. Our study also confirmed previous results on the importance of considering comorbidities, such as obesity and hypertension, as well as depression in association with post-surgical pain. Further research involving larger patient cohorts is required to validate our findings on the importance of preoperative examination of gene expression for the prognosis of the post-surgical pain development in order to provide the best possible care for patients with end-stage OA, undergoing joint replacement surgery.

## Figures and Tables

**Table 1:** Clinical and biophychosocial indices of the examined subsets of patients with end-stage OA before surgery.

Indices	Patients Who Developed Pain 6 Months after Surgery N = 17	Pain-Free Patients 6 Months after Surgery N = 33	*p*-Value (Mann-Whitney U-Test)
Patients age, years	70 (64;75)	68 (61;72)	0.70
Gender males females	3 (18%) 14 (86%)	10 (30%) 23 (70%)	0.36
Average Kellgren & Lowrence radiological stage			
III	12 (71%)	23 (70%)	0.94
IV	5 (29%)	10 (30%)	0.94
BMI, kg/m^2^	32.4 (27.1;34.5)	29.1 (27.3;32.1)	0.31
Disease duration, years	8 (5.5;12)	10 (5;14)	0.90
Erythrocyte Sedimentation Rate (ESR), mm/h	8.5 (5.5;19)	12 (10;25)	0.27
Pain (VAS), mm	60 (60;70)	70 (60;70)	0.89
DN4 score	2 (1.5;3)	2 (1;2)	0.24
PainDETECT score	7 (4;9)	4 (2;9)	0.28
HADS anxiety score	7 (4.5;9.5)	5.5 (3.5;7.5)	0.07
HADS depression score	8.5 (6;10.5)	7 (6;9)	0.25
BPI pain severity score	4.5 (3.9;5.4)	5.5 (4.5;5.8)	0.07
Total WOMAC, mm	1130 (1020;1260)	1150 (950;1200)	0.99
Total pain	230 (195;260)	230 (205;270)	0.72
Total stiffness	100 (75;110)	100 (80;115)	0.65
Total physical function	820 (710;855)	760 (685;830)	0.48
Comorbidities			
Healthy weight, (BMI 18.29–24.9 kg/m^2^)	1 (6%)	2 (6%)	1.00
Overweight (BMI 25.0–29.9 kg/m^2^)	5 (29%)	17 (51.5%)	0.14
Obesity Class I (BMI 30.0–34.9 kg/m^2^)	10 (59%)	11 (33.5%)	0.08
Obesity Class II (BMI 35.0–39.9 kg/m^2^)	1 (6%)	3 (9%)	0.71
Arterial hypertension (%)	65	30	<0.01
Cardiovascular disease (%)	6	15	0.35

**Table 2:** Gene expression data in the examined subsets of patients with end-stage OA before surgery.

Genes	Gene Expression in Patients Who Developed Pain 6 Months after Surgery N = 17	Gene Expression in Pain-Free Patients 6 Months after Surgery N = 33	*p*-Value (Mann-Whitney U-Test)
Cathepsin S	19.5(10.5;35.9)	7.2(2.5;11.1)	0.0001
Cathepsin K	11.8(4.5;33.5)	4.6(2.2;9.6)	0.005
Caspase 3	11.2(3.9;24.1)	3.6(1.3;11.5)	0.008
TIMP1	12.4(8.2;27.5)	7.6(2.4;13.0)	0.005
TNFα	4.2(1.8;7.3)	1.1(0.7;2.2)	0.01
IL-1β	5.9(1.6;10.5)	1.6(.3;3.5)	0.002
MMP-9	18.0(10.0;38.9)	12.0(5.8;28.2)	0.28
COX-2	1.1(0.5;2.7)	1.3(0.5;2.5)	0.75

**Table 3:** Correlation coefficients and their significance (*p)* between clinical and biophychosocial indices measured before surgery in patients with end-stage OA (n = 50).

	Pain at Night (VAS), mm	Total Pain	Total Physical Function	Total WOMAC	DN4 Scores	HADS Depression Scores
K&L radiological stage	0.442 *p* = 0.04		0.512 *p* = 0.01			
PainDETECT scores	0.525 *p* = 0.01				0.708 *p* < 0.01	0.546 *p* = 0.03
HADS anxiety scores			0.551 *p* = 0.01	0.472 *p* = 0.03		0.678 *p* < 0.01
HADS depression scores					0.312 *p* = 0.03	
BPI pain severity scores		0.628 *p* < 0.01	0.541 *p* = 0.01	0.590 *p* < 0.01		
